# Supplementary material for: The Impact of Perioperative Events on Cancer Recurrence and Metastasis in Patients after Radical Gastrectomy: A Review
Source: Cancers (Basel). 2022 Jul 19;14(14):3496. doi: 10.3390/cancers14143496 (PMC9319233; doi:10.3390/cancers14143496)
Supplement: Supplementary file 1 [file cancers-14-03496-s001.zip › cancers-1794417-supplementary.pdf]

Supplementary Table S1. Clinical trials comparing effects of different surgical approaches on long-term survival of patients with gastric cancer.

Trial	Year	Country	Intervention	cStage	Cases	POCs	Early mortality	Survival	Subgroup or *post hos* analysis
MRC ST-01 [1,2]	1987-1994	The United Kingdom	D1 vs. D2	I-III	200/200	28%/46%*	6.5%/13%*	OS:<br>HR=1.10 (0.87–1.39)<br>RFS:<br>HR=1.03 (0.82–1.29)	OS:<br>Pancreatico-splenectomy: HR=1.53 (1.17–2.01)
Dutch D1/D2 [3,4]	1989-1993	Netherlands	D1 vs. D2	M0	380/331	25%/43%*	4%/10%*	OS:<br>HR=1.09 (0.94-1.27)<br>Relapse:<br>HR=0.84 (0.65-1.09)	Mean OS (mouth):<br>Splenectomy/spleen preservation:<br>D1: 5.14 (3.16-7.12)/7.37 (6.68-8.06) *<br>D2: 5.19 (4.07-6.31)/9.09 (8.09-10.08) *<br>Pancreatectomy/pancreas preservation:<br>D1: 2.34 (0.00-5.23)/7.27 (6.61-7.93) *<br>D2: 4.85 (3.64-6.07)/8.81 (7.87-9.73) *
JCOG9501 [5,6]	1995-2001	Japan	D2 vs. D2 plus PAND	T2b–4, M0, CY0	263/260	20.9%/28.1%	0.8%/0.8%	OS:<br>HR=1.03 (0.78-1.38)<br>RFS:<br>HR=1.08 (0.83-1.42)	OS:<br>N+: HR=1.39 (1.02-1.89)<br>T3/T4: better in D2 only group (*P*=0.004)
JCOG0110 [7]	2002-2009	Japan	without vs. with splenectomy	Proximal T2-T4	251/254	16.1%/30.3%*	0.8%/0.4%	OS:<br>HR=0.88 (0.67-1.16)<br>RFS:<br>HR=0.87 (0.65-1.17	OS:<br>Middle and lower: HR=0.27 (0.10-0.74)<br>Splenectomy: more peritoneal disease; decreases survival in deeper tumors and with lymph node metastasis<br>7/8 patients with No.10+ died of recurrence
Yu[8]	1995-1999	Korea	without vs. with splenectomy	I-IV	103/104	8.7%/15.4%	1.0%/1.9%	5-y OS: 48.8%/54.8%	No.10+ 5-y OS: 0/0
JCOG9502 [9,10]	1995-2003	Japan	LTA vs. TH	esophageal invasion ≤ 3 cm, cT2–4	85/82	49%/34%	2.4%/0	OS:<br>HR=1.42 (0.98-2.05)	Type III tumors treated by LTA:<br>OS was lower<br>More peritoneal seeding

JCOG1001 [11]	2010-2015	Japan	Bursectomy vs. omentectomy alone	cT3-T4b	600/601	Grade 3-4: 13%/11%	0.2%/0.8%	OS: HR=1.05 (0.81-1.37) RFS: HR=1.07 (0.86-1.33)	NA
JCOG0912 [12]	2010-2013	Japan	LDG vs. ODG	I	462/459	Grade 3-4: 2%/4%	0/0	OS: HR=0.83 (0.49-1.40) RFS: HR=0.84 (0.56-1.27)	NA
KLASS-01 [13,14]	2006-2010	Korea	LDG vs. ODG	I	673/680	13.0%/19.9%*	0.6%/0.3%	5-y OS: 94.2%/93.3% 5-y CSS: 97.1%/97.2%	NA
CLASS-01 [15,16]	2012-2014	China	LDG vs. ODG	II-IVa	519/520	15.2%/12.9%	0.4%/0	DFS: HR=1.10 (0.84-1.43) OS: HR=1.19 (0.87-1.64)	NA
KLASS-02 [17]	2011-2015	Korea	LDG vs. ODG	T2-4a	492/482	15.7%/23.4%*	NA	3-y OS: 90.6%/90.3% RFS: HR=1.02 (0.75-1.39)	NA

*Statistically significant, CSS: cancer-specific survival, HR: hazard ratio, LDG: laparoscopic distal gastrectomy, LTA: left thoraco-abdominal, NA: not applicable, ODG: open distal gastrectomy, OS: overall survival, PAND: para-aortic lymph node dissection, POCs: postoperative complications, RFS: relapse-free survival, TH: transhiatal.

Supplementary Table S2. Studies on effects of different anesthetic approaches and drugs on long-term survival of patients with gastric cancer.

Author	Year	Country	Design	Intervention	Cases	Outcomes
Hiller [18]	2014	Australia	Retrospective	GA–EA vs. GA	48/21	2-y time to cancer recurrence: HR=1.19 (0.43-3.33) Epidural-36h: HR=0.33 (0.17–0.63) 2y OS: HR=1.45 (0.40-5.26) Epidural-36 h: HR=0.42 (0.21–0.83)

Cummings [19]	2014	USA	Retrospective	GA–EA vs. GA	766/1979	OS: HR=0.93 (0.84-1.03)<br>Recurrence: HR=1.40 (0.96-2.05)
Wang [20]	2016	China	Retrospective	GA–EA vs. GA	157/116	OS: HR=0.92 (0.71-1.19);<br>Younger than 65 years: improved OS (*P*=0.035)
Wang [21]	2017	China	Retrospective	GA–EA vs. GA	1362/2856	OS: HR=0.70 (0.63-0.77)
Shin [22]	2017	Korea	Retrospective	Epidural vs. IVPCA	3425/374	Recurrence: HR=1.092 (0.859–1.388)<br>Mortality: HR=0.695 (0.429–1.125)
Pei [23]	2020	China	Retrospective	GA–EA vs. GA	97/97	5-y OS: 56.7%/47.9% (*P*=0.147)
Zheng [24]	2018	China	Retrospective	propofol-based TIVA vs. sevoflurane-based INHA	897/897	OS: HR=0.65 (0.56-0.75)
Oh [25]	2019	Korea	Retrospective	propofol-based TIVA vs. remifentanil-based INHA	769/769	1-y overall mortality: HR=0.92 (0.52-1.64)<br>1y cancer-related mortality: HR=0.91 (0.50-1.67)
Huang [26]	2020	Taiwan China	Retrospective	Propofol-based TIVA vs. desflurane-based INHA	167/167	OS: HR=0.56 (0.41–0.78)

EA: epidural anesthesia, GA: general anesthesia, HR: hazard ratio, INHA: inhalation anesthesia, IVPCA: intravenous patient-controlled analgesia, OS: overall survival, TIVA, total intravenous anesthesia.

Supplementary Table S3. Meta-analyses of postoperative complications and prognosis of patients with gastric cancer.

Author	Year	Country	Number of included studies	Case	OS	CSS	RFS
Wang [27]	2019	China	16	12065	Any:<br>HR=1.79 (1.39-2.30)<br>HR=1.40 (1.06-1.84)*<br>Infectious:<br>HR=1.86 (1.22-2.83)<br>HR=1.47 (0.90-2.40)*<br>Anastomotic:<br>HR=2.02 (1.02-4.00)<br>HR=1.64 (0.78-3.46)*		Any:<br>HR=1.28 (1.10-1.49)<br>HR=1.33 (1.09-1.63)*<br>Infectious:<br>HR=1.65 (1.25-2.18)<br>HR=1.46 (1.05-2.03)*
Li [28]	2020	China	64	46198	Any:	Any:	Any:

	HR=1.58 (1.37-1.82)<br>≥ CD 2:<br>HR=1.26 (1.02-1.56)<br>Major:<br>HR=1.52 (1.23-1.88)<br>Infectious:<br>HR=1.46 (1.22-1.75)<br>Anastomotic:<br>HR=2.35 (1.68-3.29)<br>Cardiopulmonary:<br>HR=1.61 (1.21-2.15)	HR=1.90 (1.19-3.02)<br>Major:<br>HR=1.29 (0.83-1.99)<br>Infectious:<br>HR=1.68 (1.28-2.20)<br>Anastomotic:<br>HR=1.87 (0.68-5.12)<br>Cardiopulmonary:<br>HR=1.46 (1.03-2.07)	HR=1.66 (1.13-2.44)<br>≥ CD 2:<br>HR=1.26 (1.03-1.54)<br>Major:<br>HR=1.60 (1.02-1.54)<br>Infectious:<br>HR=1.47 (1.14-1.91)<br>Cardiopulmonary:<br>HR=1.07 (0.56-2.01)

*excluding in-hospital deaths, CD: Clavien-Dindo, CSS: cancer-specific survival, HR: hazard ratio, OS: overall survival, RFS: relapse-free survival.

Supplementary Table S4. Studies on effects of anemia on long-term survival of patients with gastric cancer.

Author	Year	Country	Cases	Cut-off (g/L)	Outcomes (univariate)	Outcomes (multivariate)
Jiang [29]	2019	China	1099	120	OS: HR=1.43 (1.18-1.74)	OS: HR=1.14 (0.93-1.41)
Liu [30]	2017	China	2163	120	OS: HR=1.24 (0.99-1.54)	OS: HR=1.24 (0.99-1.54)
Wang [31]	2017	China	859	NA	OS: HR=2.06 (1.68-2.52)<br>DFS: HR=1.89 (1.53-2.33)	OS: HR=1.29 (1.04-1.59)<br>DFS: NS
Chen [32]	2017	China	292	126	OS: HR=1.03 (0.68-1.56)<br>DFS: HR=1.07 (0.71-1.62)	OS: NS<br>DFS: NS
Xue [33]	2017	China	269	108	OS: *P*=0.033	OS: HR=1.02 (0.68-1.53)
Wu [34]	2017	China	210	60	DFS: *P*=0.755	DFS: HR=1.03 (0.94-1.12)
Lee [35]	2017	Korea	309	120	OS: HR=0.12 (0.05-0.31)<br>RFS: HR=0.25 (0.13-0.48)	OS: HR=0.32 (0.11-0.87)<br>RFS: HR=1.24 (0.49-3.11)
Eo [36]	2015	Korea	299	Continuous	OS: HR=0.40 (0.22-0.73)<br>DFS: HR=0.32 (0.19-0.56)	OS: NS<br>DFS: NS

Rausei [37]	2013	Italy	224	120	OS: *P*=0.113	NA
Mohri [38]	2010	Japan	357	120	OS: HR=1.81 (1.23-2.65)	NS
Shen [39]	2005	Korea	1688	120	OS: NS	OS: *P*=0.208

DFS, disease-free survival, HR: hazard ratio, NA: not applicable, NS: not significant, OS: overall survival, RFS: relapse-free survival

Supplementary Table S5. Studies on effects of intraoperative blood loss on long-term survival of patients with gastric cancer.

Author	Year	Country	Design	Cases	Stage	IBL	Outcomes	ABT adjusted	POCs adjusted
Misawa [40]	2021	Japan	Retrospective	1203	cT3-cT4b	285 (range 0-3068) ml	RFS:<br>< 200 ml: reference<br>200-400 ml: HR=1.46 (1.09-1.96)<br>> 400 ml: HR=1.52 (1.11-2.08)	Yes	No
Hayashi [41]	2020	Japan	Retrospective	115	ycStage I-III	711.5 ml	DFS:<br>< 990 ml: reference<br>> 990 ml: HR=2.40 (1.26-4.55)	Yes	Yes
Tamagawa [42]	2020	Japan	Retrospective	122	Borrmann Type IV	394 (5-1920) ml	OS:<br>< 400 ml: reference<br>≥ 400 ml: HR=1.64 (1.0-2.67);<br>5-year RFS:<br>2.1%/0*	No	No
Zhao [43]	2019	China	Retrospective	1699	cStage I-III	250(30-1500) ml	DFS:<br>< 400 ml: reference<br>≥ 400 ml: HR=1.02 (0.88-1.19)	Yes	Yes
Ito [44]	2019	Japan	Retrospective	507	pstage II–III	242 (0-2400) ml	DFS:<br>< 330 ml: reference<br>≥ 330 ml: HR=1.45 (1.01-2.09)	Excluded	Yes
Mizino [45]	2016	Japan	Retrospective	203	pstage II-III	285 (range 3-1285) ml	OS:<br>Continuous: HR=1.72 (1.03-2.87)	Excluded	Yes

Ishino [46]	2014	Japan	Retrospective	214	Stage I-II	119 (range, 4-1980) ml	OS:<br>< 1%: reference<br>≥1%: HR=14.21 (1.83-110.37)	Excluded	No
Liang [47]	2013	China	Retrospective	845	stageⅠ-III	NA	OS:<br>< 200 ml: %<br>200-400 ml: HR=1.24 (1.01-1.52);<br>> 400 ml: HR=11.59 (1.14-2.22)	Yes	Yes

*Statistically significant, DFS, disease-free survival, HR: hazard ratio, IBL: intraoperative blood loss, OS: overall survival, RFS: relapse-free survival.

Supplementary Table S6. Meta-analyses of perioperative blood transfusion and prognosis of gastric cancer.

Author	Year	Country	Number of included studies	Case	Rate (%)	Complications	Prognosis
Agnes [48]	2018	Italy	38	21485	28.5	OR=3.33 (2.10-5.29)	OS: HR1.49 (1.32-1.69)<br>DFS: HR=1.48 (1.18-1.86)<br>DSS: HR=1.66 (1.5-2.19)
Sun [49]	2015	China	18	9120	36.3	NA	All-cause mortality: OR=2.17 (1.72-2.74)<br>Cancer-related mortality: OR=2.57 (1.24-5.34)<br>recurrence: OR=1.52 (1.08-2.15)
Li [50]	2015	China	16	9189	44.8	RR=1.36 (1.02-1.81)	5-y OS: RR=0.74 (0.69-0.79)<br>Recurrence: RR=1.82 (1.32-1.51)

DFS, disease-free survival, DSS: disease-specific survival, HR: hazard ratio, NA: not applicable, OR: odds ratio, OS: overall survival, RFS: relapse-free survival, RR: risk ratio.

References

1. Cuschieri, A.; Fayers, P.; Fielding, J.; Craven, J.; Bancewicz, J.; Joypaul, V.; Cook, P. Postoperative morbidity and mortality after D1 and D2 resections for gastric cancer: preliminary results of the MRC randomised controlled surgical trial. The Surgical Cooperative Group. *Lancet* **1996**, *347,* 995-999.
2. Cuschieri, A.; Weeden, S.; Fielding, J.; Bancewicz, J.; Craven, J.; Joypaul, V.; Sydes, M.; Fayers, P. Patient survival after D1 and D2 resections for gastric cancer: long-term results of the MRC randomized surgical trial. Surgical Co-operative Group. *Br. J. Cancer* **1999**, *79,* 1522-1530.

3. Bonenkamp, J.J.; Hermans, J.; Sasako, M.; van de Velde, C.J.; Welvaart, K.; Songun, I.; Meyer, S.; Plukker, J.T.; Van Elk, P.; Obertop, H.; et al. Extended lymph-node dissection for gastric cancer. *N. Engl. J. Med.* **1999**, *340,* 908-914.
4. Songun, I.; Putter, H.; Kranenbarg, E.M.; Sasako, M.; van de Velde, C.J. Surgical treatment of gastric cancer: 15-year follow-up results of the randomised nationwide Dutch D1D2 trial. *Lancet Oncol.* **2010**, *11,* 439-449.
5. Sasako, M.; Sano, T.; Yamamoto, S.; Kurokawa, Y.; Nashimoto, A.; Kurita, A.; Hiratsuka, M.; Tsujinaka, T.; Kinoshita, T.; Arai, K.; et al. D2 lymphadenectomy alone or with para-aortic nodal dissection for gastric cancer. *N. Engl. J. Med.* **2008**, *359,* 453-462.
6. Sano, T.; Sasako, M.; Yamamoto, S.; Nashimoto, A.; Kurita, A.; Hiratsuka, M.; Tsujinaka, T.; Kinoshita, T.; Arai, K.; Yamamura, Y.; et al. Gastric cancer surgery: morbidity and mortality results from a prospective randomized controlled trial comparing D2 and extended para-aortic lymphadenectomy--Japan Clinical Oncology Group study 9501. *J. Clin. Oncol.* **2004**, *22,* 2767-2773.
7. Sano, T.; Sasako, M.; Mizusawa, J.; Yamamoto, S.; Katai, H.; Yoshikawa, T.; Nashimoto, A.; Ito, S.; Kaji, M.; Imamura, H.; et al. Randomized controlled trial to evaluate splenectomy in total gastrectomy for proximal gastric carcinoma. *Ann. Surg.* **2017**, *265,* 277-283.
8. Yu, W.; Choi, G.S.; Chung, H.Y. Randomized clinical trial of splenectomy versus splenic preservation in patients with proximal gastric cancer. *Br. J. Surg.* **2006**, *93,* 559-563.
9. Kurokawa, Y.; Sasako, M.; Sano, T.; Yoshikawa, T.; Iwasaki, Y.; Nashimoto, A.; Ito, S.; Kurita, A.; Mizusawa, J.; Nakamura, K. Ten-year follow-up results of a randomized clinical trial comparing left thoracoabdominal and abdominal transhiatal approaches to total gastrectomy for adenocarcinoma of the oesophagogastric junction or gastric cardia. *Br. J. Surg.* **2015**, *102,* 341-348.
10. Sasako, M.; Sano, T.; Yamamoto, S.; Sairenji, M.; Arai, K.; Kinoshita, T.; Nashimoto, A.; Hiratsuka, M. Left thoracoabdominal approach versus abdominal-transhiatal approach for gastric cancer of the cardia or subcardia: a randomised controlled trial. *Lancet Oncol.* **2006**, *7,* 644-651.
11. Kurokawa, Y.; Doki, Y.; Mizusawa, J.; Terashima, M.; Katai, H.; Yoshikawa, T.; Kimura, Y.; Takiguchi, S.; Nishida, Y.; Fukushima, N.; et al. Bursectomy versus omentectomy alone for resectable gastric cancer (JCOG1001): a phase 3, open-label, randomised controlled trial. *Lancet Gastroenterol. Hepatol.* **2018**, *3,* 460-468.
12. Katai, H.; Mizusawa, J.; Katayama, H.; Morita, S.; Yamada, T.; Bando, E.; Ito, S.; Takagi, M.; Takagane, A.; Teshima, S.; et al. Survival outcomes after laparoscopy-assisted distal gastrectomy versus open distal gastrectomy with nodal dissection for clinical stage IA or IB gastric cancer (JCOG0912): a multicentre, non-inferiority, phase 3 randomised controlled trial. *Lancet Gastroenterol. Hepatol.* **2020**, *5,* 142-151.
13. Kim, H.H.; Han, S.U.; Kim, M.C.; Kim, W.; Lee, H.J.; Ryu, S.W.; Cho, G.S.; Kim, C.Y.; Yang, H.K.; Park, D.J.; et al. Effect of laparoscopic distal gastrectomy vs open distal gastrectomy on long-term survival among patients with stage I gastric cancer: The KLASS-01 randomized clinical trial. *JAMA Oncol.* **2019**, *5,* 506-513.
14. Kim, W.; Kim, H.H.; Han, S.U.; Kim, M.C.; Hyung, W.J.; Ryu, S.W.; Cho, G.S.; Kim, C.Y.; Yang, H.K.; Park, D.J.; et al. Decreased morbidity of laparoscopic distal gastrectomy compared with open distal gastrectomy for stage I gastric cancer: Short-term outcomes from a multicenter randomized controlled trial (KLASS-01). *Ann. Surg.* **2016**, *263,* 28-35.
15. Yu, J.; Huang, C.; Sun, Y.; Su, X.; Cao, H.; Hu, J.; Wang, K.; Suo, J.; Tao, K.; He, X.; et al. Effect of laparoscopic vs open distal gastrectomy on 3-year disease-free survival in patients with locally advanced gastric cancer: The CLASS-01 randomized clinical trial. *JAMA* **2019**, *321,* 1983-1992.
16. Hu, Y.; Huang, C.; Sun, Y.; Su, X.; Cao, H.; Hu, J.; Xue, Y.; Suo, J.; Tao, K.; He, X.; et al. Morbidity and mortality of laparoscopic versus open D2 distal gastrectomy for advanced

gastric cancer: a randomized controlled trial. *J. Clin. Oncol.* **2016**, *34,* 1350-1357.

17. Hyung, W.J.; Yang, H.K.; Park, Y.K.; Lee, H.J.; An, J.Y.; Kim, W.; Kim, H.I.; Kim, H.H.; Ryu, S.W.; Hur, H.; et al. Long-term outcomes of laparoscopic distal gastrectomy for locally advanced gastric cancer: The KLASS-02-RCT randomized clinical trial. *J. Clin. Oncol.* **2020**, *38,* 3304-3313.
18. Hiller, J.G.; Hacking, M.B.; Link, E.K.; Wessels, K.L.; Riedel, B.J. Perioperative epidural analgesia reduces cancer recurrence after gastro-oesophageal surgery. *Acta. Anaesthesiol. Scand.* **2014**, *58,* 281-290.
19. Cummings, K.C. 3rd; Patel, M.; Htoo, P.T.; Bakaki, P.M.; Cummings, L.C.; Koroukian, S. A comparison of the effects of epidural analgesia versus traditional pain management on outcomes after gastric cancer resection: a population-based study. *Reg. Anesth. Pain Med.* **2014**, *39,* 200-207.
20. Wang, J.; Guo, W.; Wu, Q.; Zhang, R.; Fang, J. Impact of combination epidural and general anesthesia on the long-term survival of gastric cancer patients: A retrospective study. *Med. Sci. Monit.* **2016**, *22,* 2379-2385.
21. Wang, Y.; Wang, L.; Chen, H.; Xu, Y.; Zheng, X.; Wang, G. The effects of intra- and post-operative anaesthesia and analgesia choice on outcome after gastric cancer resection: a retrospective study. *Oncotarget* **2017**, *8,* 62658-62665.
22. Shin, S.; Kim, H.I.; Kim, N.Y.; Lee, K.Y.; Kim, D.W.; Yoo, Y.C. Effect of postoperative analgesia technique on the prognosis of gastric cancer: a retrospective analysis. *Oncotarget* **2017**, *8,* 104594-104604.
23. Pei, J.P.; Zhang, C.D.; Liang, Y.; Zhang, C.; Wu, K.Z.; Zhao, Z.M.; Dai, D.Q. Effects of epidural combined with general anesthesia versus general anesthesia alone in gastric cancer surgery: a propensity score matching analysis. *Ann. Transl. Med.* **2020**, *8,* 473.
24. Zheng, X.; Wang, Y.; Dong, L.; Zhao, S.; Wang, L.; Chen, H.; Xu, Y.; Wang, G. Effects of propofol-based total intravenous anesthesia on gastric cancer: a retrospective study. *Onco. Targets. Ther.* **2018**, *11,* 1141-1148.
25. Oh, T.K.; Kim, H.H.; Jeon, Y.T. Retrospective analysis of 1-year mortality after gastric cancer surgery: Total intravenous anesthesia versus volatile anesthesia. *Acta. Anaesthesiol. Scand.* **2019**, *63,* 1169-1177.
26. Huang, N.C.; Lee, M.S.; Lai, H.C.; Lin, H.T.; Huang, Y.H.; Lu, C.H.; Hsu, C.H.; Wu, Z.F. Propofol-based total intravenous anesthesia improves survival compared to desflurane anesthesia in gastric cancer surgery: A retrospective analysis. *Medicine (Abingdon)* **2020**, *99,* e20714.
27. Wang, S.; Xu, L.; Wang, Q.; Li, J.; Bai, B.; Li, Z.; Wu, X.; Yu, P.; Li, X.; Yin, J. Postoperative complications and prognosis after radical gastrectomy for gastric cancer: a systematic review and meta-analysis of observational studies. *World J. Surg. Oncol.* **2019**, *17,* 52.
28. Li, J.; Zhang, Y.; Hu, D.M.; Gong, T.P.; Xu, R.; Gao, J. Impact of postoperative complications on long-term outcomes of patients following surgery for gastric cancer: A systematic review and meta-analysis of 64 follow-up studies. *Asian J. Surg.* **2020**, *43,* 719-729.
29. Jiang, J.; Ouyang, J.; Liu, S.; Chen, J.; Zhang, H.; Wang, C.; Wu, W.; Zhang, C.; He, Y. The prognostic impact of pretreatment anemia in patients with gastric cancer and nonhypoalbuminemia undergoing curative resection: a retrospective study. *Ann. Transl. Med.* **2021**, *9,* 1046.
30. Liu, X.; Qiu, H.; Huang, Y.; Xu, D.; Li, W.; Li, Y.; Chen, Y.; Zhou, Z.; Sun, X. Impact of preoperative anemia on outcomes in patients undergoing curative resection for gastric cancer: a single-institution retrospective analysis of 2163 Chinese patients. *Cancer Med.* **2018**, *7,* 360-369.

31. Wang, S.L.; Ma, L.L.; Chen, X.Y.; Zhou, D.L.; Li, B.; Huang, D.D.; Yu, Z.; Shen, X.; Zhuang, C.L. Impact of visceral fat on surgical complications and long-term survival of patients with gastric cancer after radical gastrectomy. *Eur. J. Clin. Nutr.* **2018**, *72,* 436-445.
32. Chen, L.; Yan, Y.; Zhu, L.; Cong, X.; Li, S.; Song, S.; Song, H.; Xue, Y. Systemic immune-inflammation index as a useful prognostic indicator predicts survival in patients with advanced gastric cancer treated with neoadjuvant chemotherapy. *Cancer Manag. Res.* **2017**, *9,* 849-867.
33. Xue, F.; Lin, F.; Yin, M.; Feng, N.; Zhang, X.; Cui, Y.G.; Yi, Y.P.; Kong, X.Y.; Chen, X.; Liu, W.Z. Preoperative albumin/globulin ratio is a potential prognosis predicting biomarker in patients with resectable gastric cancer. *Turk. J. Gastroenterol*. **2017**, *28,* 439-445.
34. Wu, G.; Zhang, D.Y.; Duan, Y.H.; Zhang, Y.Q.; Cui, X.N.; Luo, Z. Correlations of hemoglobin level and perioperative blood transfusion with the prognosis of gastric cancer: A retrospective study. *Med. Sci. Monit.* **2017**, *23,* 2470-2478.
35. Lee, J.W.; Lee, M.S.; Chung, I.K.; Son, M.W.; Cho, Y.S.; Lee, S.M. Clinical implication of FDG uptake of bone marrow on PET/CT in gastric cancer patients with surgical resection. *World J. Gastroenterol.* **2017**, *23,* 2385-2395.
36. Eo, W.K.; Jeong, D.W.; Chang, H.J.; Won, K.Y.; Choi, S.I.; Kim, S.H.; Chun, S.W.; Oh, Y.L.; Lee, T.H.; Kim, Y.O.; et al. Absolute monocyte and lymphocyte count prognostic score for patients with gastric cancer. *World J. Gastroenterol.* **2015**, *21,* 2668-2676.
37. Rausei, S.; Ruspi, L.; Galli, F.; Tirotta, F.; Inversini, D.; Frattini, F.; Chiappa, C.; Rovera, F.; Boni, L.; Dionigi, G.; et al. Peri-operative blood transfusion in gastric cancer surgery: prognostic or confounding factor. *Int. J. Surg.* **2013**, *11 Suppl 1,* S100-103.
38. Mohri, Y.; Tanaka, K.; Ohi, M.; Yokoe, T.; Miki, C.; Kusunoki, M. Prognostic significance of host- and tumor-related factors in patients with gastric cancer. *World J. Surg.* **2010**, *34,* 285-290.
39. Shen, J.G.; Cheong, J.H.; Hyung, W.J.; Kim, J.; Choi, S.H.; Noh, S.H. Pretreatment anemia is associated with poorer survival in patients with stage I and II gastric cancer. *J. Surg. Oncol.* **2005**, *91,* 126-130.
40. Misawa, K.; Kurokawa, Y.; Mizusawa, J.; Takiguchi, S.; Doki, Y.; Makino, S.; Choda, Y.; Takeno, A.; Tokunaga, M.; Sano, T.; et al. Negative impact of intraoperative blood loss on long-term outcome after curative gastrectomy for advanced gastric cancer: exploratory analysis of the JCOG1001 phase III trial. *Gastric Cancer* **2021** *25,* 459-467.
41. Hayashi, M.; Yoshikawa, T.; Yura, M.; Otsuki, S.; Yamagata, Y.; Morita, S.; Katai, H.; Nishida, T. Intraoperative blood loss as an independent prognostic factor for curative resection after neoadjuvant chemotherapy for gastric cancer: a single-center retrospective cohort study. *Surg. Today* **2021**, *51,* 293-302.
42. Tamagawa, H.; Aoyama, T.; Kano, K.; Numata, M.; Atsumi, Y.; Hara, K.; Kazama, K.; Koumori, K.; Murakawa, M.; Hashimoto, I.; et al. The impact of intraoperative blood loss on the long-term prognosis after curative resection for borrmann type IV gastric cancer: A retrospective multicenter study. *Anticancer Res.* **2020**, *40,* 405-412.
43. Zhao, B.; Huang, X.; Lu, H.; Zhang, J.; Luo, R.; Xu, H.; Huang, B. Intraoperative blood loss does not independently affect the survival outcome of gastric cancer patients who underwent curative resection. *Clin. Transl. Oncol.* **2019**, *21,* 1197-1206.
44. Ito, Y.; Kanda, M.; Ito, S.; Mochizuki, Y.; Teramoto, H.; Ishigure, K.; Murai, T.; Asada, T.; Ishiyama, A.; Matsushita, H.; et al. Intraoperative blood loss is associated with shortened postoperative survival of patients with stage II/III gastric cancer: Analysis of a multi-institutional dataset. *World J. Surg.* **2019**, *43,* 870-877.
45. Mizuno, A.; Kanda, M.; Kobayashi, D.; Tanaka, C.; Iwata, N.; Yamada, S.; Fujii, T.; Nakayama, G.; Sugimoto, H.; Koike, M.; et al. Adverse effects of intraoperative blood loss on

long-term outcomes after curative gastrectomy of patients with stage II/III gastric cancer. *Dig. Surg.* **2016**, *33,* 121-128.

46. Ishino, Y.; Saigusa, S.; Ohi, M.; Yasuda, H.; Tanaka, K.; Toiyama, Y.; Mohri, Y.; Kusunoki, M. Preoperative C-reactive protein and operative blood loss predict poor prognosis in patients with gastric cancer after laparoscopy-assisted gastrectomy. *Asian J. Endosc. Surg.* **2014**, *7,* 287-294.

47. Liang, Y.X.; Guo, H.H.; Deng, J.Y.; Wang, B.G.; Ding, X.W.; Wang, X.N.; Zhang, L.; Liang, H. Impact of intraoperative blood loss on survival after curative resection for gastric cancer. *World J. Gastroenterol.* **2013**, *19,* 5542-5550.

48. Agnes, A.; Lirosi, M.C.; Panunzi, S.; Santocchi, P.; Persiani, R.; D' Ugo, D. The prognostic role of perioperative allogeneic blood transfusions in gastric cancer patients undergoing curative resection: A systematic review and meta-analysis of non-randomized, adjusted studies. *Eur. J. Surg. Oncol.* **2018**, *44,* 404-419.

49. Sun, C.; Wang, Y.; Yao, H.S.; Hu, Z.Q. Allogeneic blood transfusion and the prognosis of gastric cancer patients: systematic review and meta-analysis. *Int. J. Surg.* **2015**, *13,* 102-110.

50. Huang, X.Z.; Yang, Y.C.; Chen, Y.; Wu, C.C.; Lin, R.F.; Wang, Z.N.; Zhang, X. Preoperative anemia or low hemoglobin predicts poor prognosis in gastric cancer patients: A meta-analysis. *Dis. Markers* **2019**, *2019,* 7606128.
